# Perception and Acceptance of Telemedicine Use in Health Care Among the General Public in China: Web-Based Cross-Sectional Survey

**DOI:** 10.2196/53497

**Published:** 2024-07-16

**Authors:** Yulan Lin, Xiaonan Xu, Yiyang Liu, Haridah Alias, Zhijian Hu, Li Ping Wong

**Affiliations:** 1 Fujian Key Laboratory of Environmental Factors and Cancer Department of Epidemiology and Health Statistics, School of Public Health Fujian Medical University Fuzhou China; 2 Centre of Population Heath (CePH) Department of Social and Preventive Medicine, Faculty of Medicine Universiti Malaya Kuala Lumpur Malaysia

**Keywords:** telemedicine, acceptance, China, general public, COVID-19, pandemic, health care, public health, health care delivery, health care services, survey, cross-sectional survey, consultation, teleconsultation, health care system

## Abstract

**Background:**

The COVID-19 pandemic is bringing about substantial changes in health care systems, leading to a significant shift toward telemedicine for the delivery of health care services.

**Objective:**

This study aims to examine the relationship between perceived usefulness and ease of use of telemedicine services and their association with the behavioral intention to use telemedicine.

**Methods:**

An anonymous cross-sectional survey was conducted in China. Partial least squares structural equation modeling was used to determine significant predictors of intention to use telemedicine consultation. Types of illnesses that favored seeking telemedicine consultation, as well as the most preferred platform for conducting telemedicine consultations, were also investigated.

**Results:**

In total, 1006 participants completed the survey. A total of 44.3% (n=446) reported being very likely and 49.3% (n=496) reported being likely to seek telemedicine consultation. Overall, the majority of participants expressed strong agreement or agreement regarding the perceived usefulness of telemedicine. Likewise, the majority indicated strong agreement or agreement when it came to their perception of the ease of using telemedicine. In the partial least squares structural equation modeling, perceived usefulness (β=0.322; *P*<.001) and perceived ease of use (β=0.118; *P*=.01) were significantly associated with a higher likelihood of seeking telemedicine consultation. A considerable number of participants expressed willingness to use telemedicine services for various medical conditions, particularly respiratory (n=340, 33.8%), skin (n=316, 31.4%), and musculoskeletal issues (n=316, 31.4%) while showing less interest in seeking telemedicine consultations for reproductive health (n=44, 4.4%) and cancer (n=64, 6.4%). The majority preferred video chat (n=443, 44%) and text chat (n=317, 31.5%) as their most preferred platforms for telemedicine consultation, while a smaller proportion preferred telephone (n=193, 19.2%) and email (n=53, 5.3%).

**Conclusions:**

Telemedicine has the potential to play a larger role in China’s health care system. The preferences for certain platforms over others may influence service design and implementation.

## Introduction

Telemedicine in China had its beginnings earlier, but the COVID-19 pandemic accelerated its widespread adoption and practice [1]. In China, before the pandemic, telemedicine was an emerging health care delivery approach that was undergoing rapid development. Telemedicine has been instrumental in enhancing the distribution of top-tier health care resources from urban centers to remote rural areas. Prior to the pandemic, it played a pivotal role in ensuring equitable health care services between urban and underserved regions while also advancing the development of China’s tiered medical system [2]. The telemedicine network in China was activated immediately following the initial COVID-19 outbreak in January 2020 [3]. The country leveraged telemedicine and tele-education effectively throughout the pandemic, ensuring the safety of both patients and health care workers while delivering timely health care services. Telemedicine proved its feasibility, acceptability, and effectiveness, leading to substantial enhancements in health care outcomes during the pandemic in China [3].

Telemedicine has indeed proven to be useful during both public health emergencies and nonemergency times, offering cost savings and maintaining quality medical care through remote consultations [4]. As the world transitions back to prepandemic normalcy, uncertainties persist regarding whether the general public’s acceptance and use of telemedicine have evolved. It remains essential for the public to sustain their backing for telemedicine services, even as the pandemic subsides. Research is warranted to assess this acceptance, as it holds significant importance, particularly in identifying areas where telemedicine can be improved, such as addressing concerns around privacy, security, and quality of care. This helps ensure that telemedicine services align with patient needs, improve health outcomes, and optimize resource use.

Perceived ease of use was reinforced as a key driver of using telemedicine [5]. Telemedicine enhances health care access by allowing practitioners to offer services beyond geographical boundaries, reducing noncritical hospital visits, and alleviating congestion in health care facilities [5]. Telemedicine also effectively bridges the gap between providers and patients in remote locations, ensuring easy access to health care services for patients in these areas [6]. Both perceived ease of use and perceived usefulness in telemedicine are important indicators for assessing the successful adoption and integration of this innovative health care approach into our modern health care systems. These 2 factors play a pivotal role in influencing individuals’ decisions to embrace telemedicine, ultimately shaping its impact on health care accessibility and efficiency.

The first aim of this study was to examine the relationship between perceived usefulness and ease of use of telemedicine services and their association with the general public’s behavioral intention to use telemedicine. Using the technology acceptance model (TAM) as a theoretical framework, we postulate that actual behaviors are predicted by behavioral intention, and behavioral intention is underpinned by perceived usefulness and perceived ease of telemedicine use. The TAM is widely used to investigate the acceptance and use of technology by individuals. It is one of the most popular frameworks for understanding technology adoption at the individual level [7]. Second, this study also explored the types of illnesses that people would prefer to seek telemedicine consultations as well as their preferred platforms for conducting these consultations. The study’s findings aim to contribute to a deeper understanding of the factors driving telemedicine adoption in China while also providing valuable insights for policy makers and health care providers looking to improve the accessibility and acceptance of telemedicine services in the aftermath of the COVID-19 pandemic.

## Methods

### Study Participants and Survey Design

A nationwide cross-sectional open survey using a web-based questionnaire was conducted from August 30, 2021, to May 1, 2022. A convenient sampling method was used. The study used Wenjuanxing (Changsha Jingwei Cloud Computing Technology Co, Ltd), a widely accepted web-based questionnaire survey platform in China for data collection. The survey platform prevents multiple responses by the same individual by disallowing users with the same IP address from accessing the survey more than once. The survey was disseminated mainly using WeChat (Tencent Holdings Ltd), China’s largest social media platform. Responses were automatically captured by the survey platform and transferred to SPSS (IBM Corp) for analysis. The inclusion criteria were that the participants were Chinese citizens aged 18 years or older. Data collection took place across 6 main regions in mainland China: North, Northeast, East, Southcentral, Southwest, and Northwest. First, the research team advertised and disseminated the survey with colleagues, students, and their network contacts. Second, the snowball sampling technique was used to enlist more participants across all provinces in mainland China, where individuals who received the survey link were encouraged to pass on the invitations to their own network members.

The calculated sample size was 385, based on a normal approximation of binomial distribution with a finite population correction applied, assuming an observer proportion of participants selecting a specific response option of 50%, a 95% confidence level, and a margin of error of 5%. The sample size was multiplied by the predicted design effect of 2 to account for the use of convenience sampling and a web-based survey [8]. Therefore, the minimum sample size for this study was 770 (385×2) participants. Survey participants were encouraged to share the survey link with everyone in their contact list.

### Measures

#### Overview

The demographic characteristics of study participants were first determined. Subsequently, the survey questionnaire (Multimedia Appendix 1) was divided into 3 sections that assessed participants’ perceptions of telemedicine services in terms of usefulness, ease of use, and behavioral intention to use them if it is available.

#### Demographic Characteristics

In this study, demographic information such as participants’ age, sex, highest educational attainment, annual family income, and birthplace were collected.

#### Perceived Usefulness of Telemedicine

The questions on perceived usefulness were self-developed to evaluate participants’ perceptions of telemedicine’s use and consist of 4 items. These questions assess the usefulness of telemedicine during public health emergencies and nonemergency times, its cost-effectiveness compared to conventional medical care-seeking methods, and its capacity to deliver quality health care services on par with traditional face-to-face consultations. The response choices were presented using a 4-point Likert scale: 4=strongly agree, 3=agree, 2=disagree, and 1=strongly disagree. The items have a Cronbach α value of 0.631, composite reliability (CR) of 0.760, and average variance extracted (AVE) of 0.391.

#### Perceived Ease of Telemedicine Use

Likewise, the perceived ease of use questionnaire was also self-developed. The questionnaire comprises 5 questions aimed at evaluating the ease with which participants perceive telemedicine as an effective way to enhance health care access, provide services across geographical boundaries, deliver care to remote patients, reduce noncritical hospital visits, and alleviate hospital congestion. Likewise, the response choices were presented using a 4-point Likert scale: 4=strongly agree, 3=agree, 2=disagree, and 1=strongly disagree. The Cronbach α value for perceived ease of telemedicine use is 0.513, with a CR of 0.731, and AVE of 0.406.

#### Behavioral Intention

The question about behavioral intention asked participants how inclined they were to seek telemedicine consultation if it were accessible to them. Option answers were “very likely,” “likely,” “unlikely,” and “strongly unlikely.” They were also asked about the types of illnesses they would like to seek telemedicine consultation as well as their most preferred platform for conducting telemedicine consultations.

All questions in the questionnaire require a response; participants must fill them out before proceeding to the next page. As a result, all responses received through the survey platform are complete. Before the survey commenced, the questionnaire underwent content validation by a panel of experts in the field and was subsequently pilot-tested with the general public before its official dissemination.

### Statistical Analyses

Partial least squares structural equation modeling (PLS-SEM) was used to predict factors influencing the likelihood of seeking telemedicine consultation if it is available. This technique assesses the reliability of the data set, the statistical significance of the coefficients, and the error of the estimated path coefficients [9]. The bootstrapped significance calculation was performed in SmartPLS software (version 3.2.8; SmartPLS GmbH). Prior to running the path model, the construct validity (convergent and discriminant) was tested. In the PLS-SEM analysis, perceived usefulness and perceived ease of use were considered as reflective constructs, and all other independent variables and the outcome—likelihood to seek telemedicine consultation if it is available—were single-item constructs. As PLS-SEM is a very useful technique for evaluating complex theoretical relationships between multiple variables, the PLS-SEM analysis is also used to explain the effect of demographic variables on the association between seeking telemedicine consultation and the perceived usefulness of telemedicine or perceived ease of telemedicine. Results of the measurement model indicated that all indicators had an acceptable outer loading (range 0.541-0.721) with a Cronbach α value of 0.513 for perceived usefulness and 0.631 for perceived ease of use, a CR value of 0.731 for perceived usefulness and 0.760 for perceived ease of use, and an AVE value of 0.406 for perceived usefulness and 0.391 for perceived ease of use. The variance inflation factors for all indicators were below 2.5 (ranging from 1.108 to 1.278), which revealed that all indicators belonging to these 2 constructs were adequately independent. Discriminant validity assessment through the heterotrait-monotrait ratio of correlations method also indicated that all heterotrait-monotrait values were lower than the most restrictive threshold (0.85; range 0.028-0.558).

### Ethical Considerations

This study was implemented according to the principles of the Declaration of Helsinki and granted ethical approval by the Fujian Medical University Research Ethics Committee, China (approval FJMU 2022 No 64). Consent was acquired by presenting the consent statement at the survey’s outset and mandating participants to click an agreement button, signifying their consent to participate, before proceeding to answer the survey questions. Informed consent involves providing participants with information sheets, informing them about the survey’s timeframe and the estimated time required to complete it, and assuring them of the anonymity of their responses. The participant’s information sheet also contains details about the investigators and the institution conducting the survey. The participants were informed that their participation was voluntary, and no incentives, whether monetary or nonmonetary, were provided. The web-based consent used in the web-based survey received approval from the ethical committee. The survey did not collect any identifying information from participants. Data are securely stored on a cloud platform accessible only to the principal investigator.

## Results

### User Statistics

**Figure 1:**
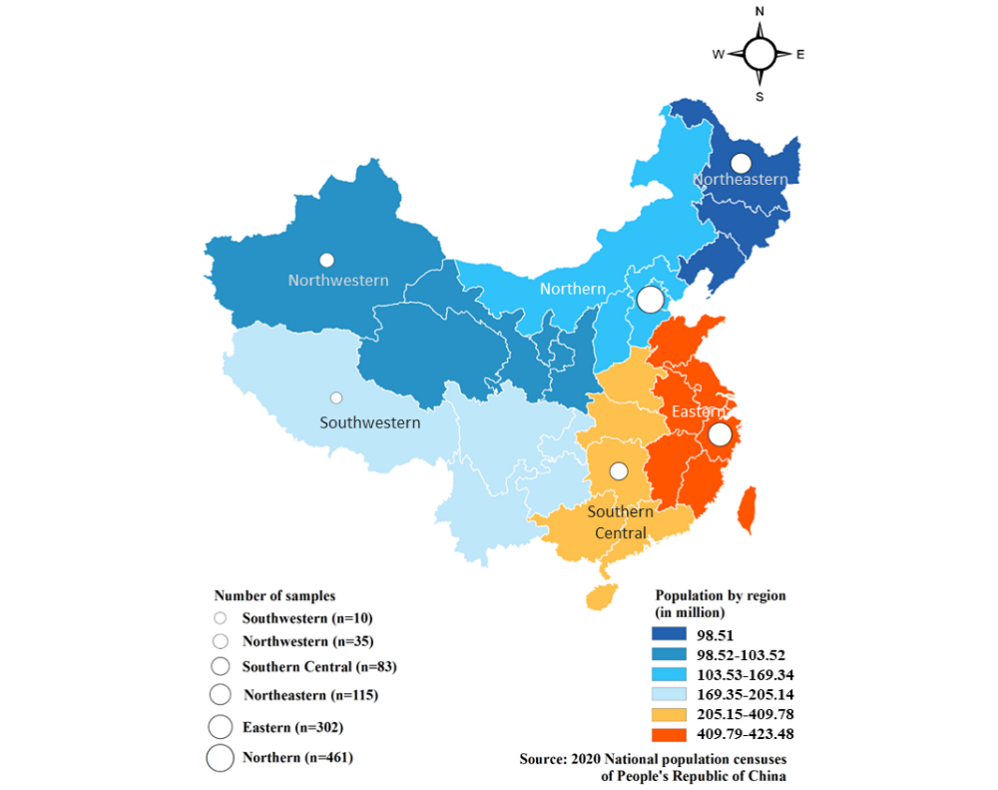
The proportion of responses by region and population distribution.

A total of 1006 complete responses were received in the survey. Table 1 shows the demographics of the study participants. The age of the participants ranged from 18 to 64 (mean 28.2, SD 7.1) years. A substantial portion of the participants fell within the 18-29 years age bracket, accounting for 64.9% (n=653) of the total. Sex distribution was nearly equal, with 50.7% (n=510) male participants and 49.3% (n=496) female participants. In terms of their highest educational attainment, 16% (n=161) had completed high school, while the majority (n=793, 78.8%) were university graduates. When considering income categories, the majority of participants reported family annual incomes in the range of CN ¥150,001 to CN ¥200,000 (approximately US $20,655 to US $27,540), comprising 53.2% (n=416) of the sample. A substantial proportion of participants (n=801, 79.6%) were from urban areas. The majority of responses were from Northern (45.8%, n=461) and Eastern (n=302, 30%) regions (Figure 1).

**Table 1:** Participants’ characteristics (N=1006).

Sociodemographic characteristics		Values, n (%)
**Age group (years)**		
	18-29	653 (64.9)
	30-39	275 (27.3)
	40-64	78 (7.8)
**Sex**		
	Male	510 (50.7)
	Female	496 (49.3)
**Highest educational level**		
	Secondary school and below	52 (5.2)
	High school or technical school	161 (16)
	Tertiary	793 (78.8)
**Family annual income (** **CN ¥; US $** **)**		
	<50,000 (<6885)	83 (8.3)
	50,000-100,000 (6885-13,770)	222 (22.1)
	100,001-150,000 (13,770-20,655)	285 (28.3)
	150,001-200,000 (20,655-27,540)	250 (24.9)
	250,001-300,000 (27,540-41,310)	130 (12.9)
	>300,000 (>41,310)	36 (3.6)
**Birthplace**		
	Urban	801 (79.6)
	Rural	205 (20.4)

### Evaluation Outcomes

Table 2 shows the proportion of responses on individual items of perceived usefulness and ease of use. The highest percentage (n=482, 47.9%) strongly agreed that telemedicine is essential in nonpublic emergency situations. In contrast, a relatively lower proportion (n=213, 21.2%) strongly agreed that telemedicine is useful during public health emergencies. However, there was a nearly equal split among participants who strongly agreed that telemedicine visits are cost-saving compared to conventional medical care–seeking methods (n=345, 34.3%) as well as those who strongly agreed that telemedicine provides medical services of equal quality to traditional face-to-face consultations (n=317, 31.5%). Overall, the majority of participants expressed strong agreement or agreement regarding the perceived usefulness of telemedicine.

Likewise, the majority of participants indicated strong agreement or agreement when it came to their perception of the ease of using telemedicine. Among all the items, the highest proportion of strong agreement was observed in the perception that telemedicine is an effective means to improve access to health services (n=427, 42.4%), followed by the belief that telemedicine can enable health care providers to deliver services to patients in remote locations, with 37.7% (n=379) strongly agreeing. A relatively lower proportion of strong agreement was noted for the statement that telemedicine can replace hospital visits for noncritical illnesses, with 31.4% (n=316) strongly agreeing.

**Figure 2:**
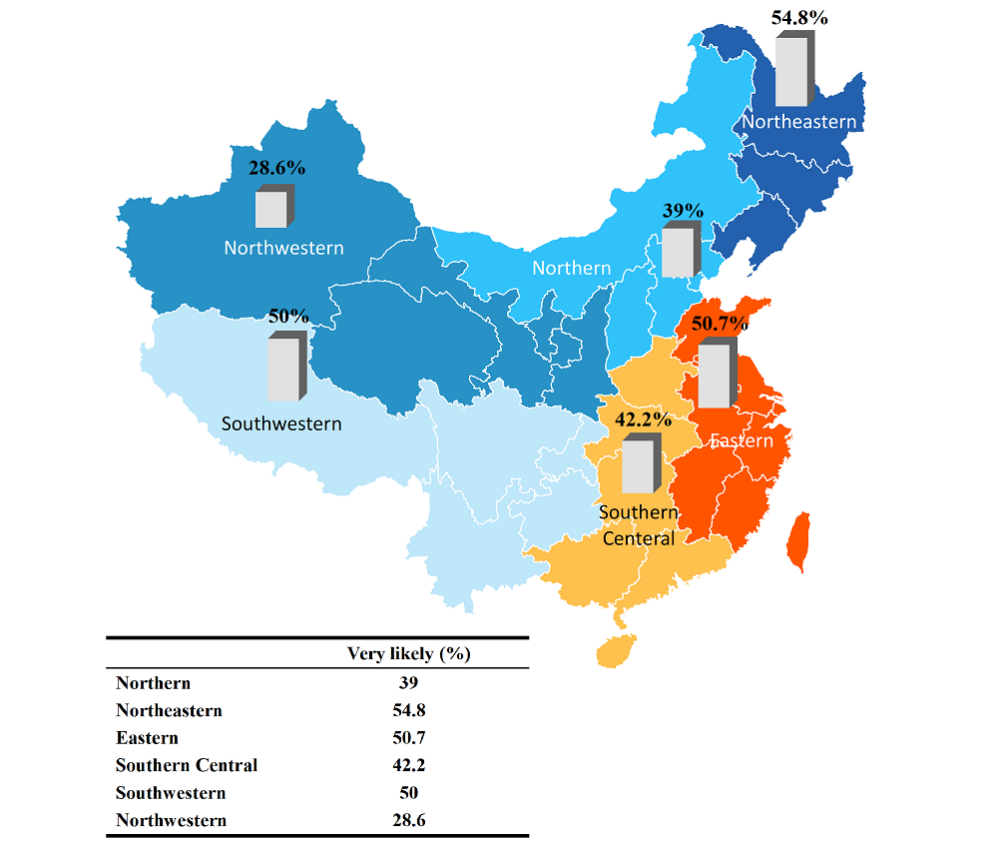
Acceptance of telemedicine consultations by region.

**Figure 3:**
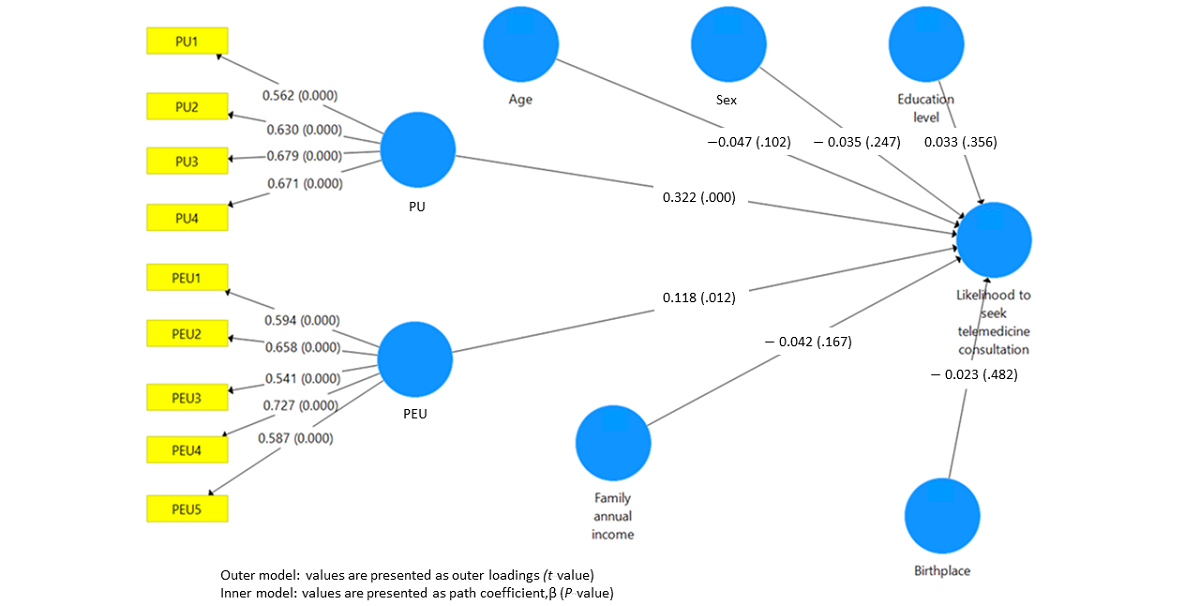
Partial least squares structural equation modeling of factors influencing the likelihood of seeking telemedicine services. PEU: perceived ease of use; PU: perceived usefulness.

Overall, a total of 44.3% (n=446) reported being very likely to seek telemedicine consultation, and 49.3% (n=496) reported being likely to seek telemedicine consultation. Only 5.2% (n=52) reported being unlikely, and 1.2% (n=12) reported being strongly unlikely. Acceptance of telemedicine consultation by region is shown in Figure 2. The Northeastern region exhibits the highest likelihood of seeking telemedicine, with 54.8% (n=153) of individuals reported very likely to seek telemedicine consultation. The PLS-SEM in Figure 3 shows the associations of all the factors associated with the likelihood of seeking telemedicine consultation. As depicted in the figure, perceived usefulness (β=0.322; *P*<.001) showed the greatest effect in influencing a higher likelihood to seek telemedicine consultation. Perceived ease of use (β=0.118; *P*=.01) is also associated with a higher likelihood to seek telemedicine consultation. Results for adjusted *R*^2^ indicated that this model explained 17.1% of the total variance in the likelihood of seeking telemedicine consultation.

Table 3 shows the responses of participants’ perspectives on the types of illnesses that they would like to seek telemedicine consultation. Only 16.4% (n=165) indicated a likelihood of seeking telemedicine for all illnesses. A high proportion of participants reported that they were likely to seek telemedicine consultation for respiratory (n=340, 33.8%), skin diseases, and musculoskeletal (n=316, 31.4%)-related diseases. However, a low proportion reported the likelihood of seeking telemedicine for reproductive health (n=44, 4.4%) and cancer (n=64, 6.4%). The findings on the most preferred platform for telemedicine consultation indicate that the majority preferred video chat (n=443, 44%), followed by text chat (n=317, 31.5%). A small proportion reported preferring telephone (n=193, 19.2%) and via email (n=53, 5.3%).

**Table 2:** Responses regarding perceived usefulness and ease of use of telemedicine services (N=1006).

Items		Strongly agree, n (%)	Agree, n (%)	Disagree, n (%)	Strongly disagree, n (%)
**PU^a^**					
	PU1: Telemedicine is essential for use during public health emergencies (eg, pandemics, infectious disease outbreaks, and flooding)	213 (21.2)	681 (67.7)	101 (10)	11 (1.1)
	PU2: Telemedicine is essential for use during the time of no public health emergencies	482 (47.9)	401 (39.9)	103 (10.2)	20 (2)
	PU3: Telemedicine visits are cost savings compared to conventional way of seeking medical care	345 (34.3)	529 (52.6)	108 (10.7)	24 (2.4)
	PU4: Telemedicine can equally provide quality medical service as the traditional face-to-face consultation	317 (31.5)	523 (52)	134 (13.3)	32 (3.2)
**PEU^b^**					
	PEU1: Telemedicine is an effective way to improve access to health services	427 (42.4)	453 (45)	102 (10.1)	24 (2.4)
	PEU2: Telehealth practitioners can provide medical services across geographic borders	360 (25.8)	506 (50.3)	116 (11.5)	24 (2.4)
	PEU3: Telemedicine can enable providers deliver health services to patients at remote locations	379 (37.7)	519 (51.6)	77 (7.7)	31 (3.1)
	PEU4: Telemedicine can circumvent hospital visits of noncritical illnesses	316 (31.4)	499 (49.6)	132 (13.1)	59 (5.9)
	PEU5: Telemedicine can help to alleviate hospital congestion	311 (30.9)	537 (53.4)	119 (11.8)	39 (3.9)

^a^PU: perceived usefulness.

^b^PEU: perceived ease of use.

**Table 3:** Telemedicine consultation preferences by type of illness (N=1006).

Type of illness	Values, n (%)
Respiratory (eg, asthma, bronchitis, and tuberculosis)	340 (33.8)
Musculoskeletal (eg, arthritis, rheumatism, and backache)	316 (31.4)
Skin diseases	316 (31.4)
Endocrine (eg, thyroid disease, diabetes, and lupus	274 (27.2)
Circulatory (hypertension)	272 (27)
Cardiovascular diseases (heart problem and stroke)	260 (25.8)
Neurologic diseases (migraine, sclerosis, and epilepsy)	259 (25.7)
Psychological (depression, anxiety, and other emotional issue)	243 (24.2)
Digestive diseases (trouble with mouth, gums, ulcers, indigestion, diarrhea, and constipation)	243 (24.2)
All illnesses	165 (16.4)
Infectious diseases	98 (9.7)
Cancer	64 (6.4)
Reproductive health and related diseases	44 (4.4)

## Discussion

### Principal Findings

This study shed light on the perceptions of both the perceived usefulness and ease of use of telemedicine among the study participants. First, in regard to findings on the perceived usefulness of telemedicine, it is noteworthy that the highest percentage of participants strongly agreed that telemedicine is essential in nonpublic emergency situations. This finding reflects a broad acknowledgment of telemedicine’s importance, even as the pandemic subsides, indicating continued public support for its use in daily health care. A considerably high proportion of participants strongly agreed that telemedicine visits are cost-saving compared to conventional medical care–seeking methods, and those who strongly agreed that telemedicine provides medical services of equal quality to traditional face-to-face consultations align with the core objectives of telemedicine implementation [10-12].

Second, with regard to the findings on the perceived ease of use of telemedicine, the majority of participants indicated strong agreement or agreement across all items related to ease of use. The highest proportion of strong agreement was observed in the perception that telemedicine is an effective means to improve access to health services. This highlights the belief that telemedicine can bridge geographical barriers and enhance health care accessibility, especially for those in remote or underserved areas. Moreover, a high proportion of participants strongly agreed that telemedicine can enable health care providers to deliver services to patients in remote locations, reaffirming the perception that telemedicine has the potential to expand the reach of health care services, even to geographically isolated communities. The existing health inequity and scarcity of medical resources in rural China provide a unique opportunity for telemedicine to play a crucial role in bridging this gap [13]. The positive perceptions of telemedicine suggest that, overall, the public is open to adopting new technologies to address their health care needs.

Finally, while a relatively lower proportion of participants strongly agreed that telemedicine can replace hospital visits for noncritical illnesses, it still signifies a significant level of acceptance. This suggests that individuals may be open to using telemedicine for a range of health care needs, albeit with some reservations about replacing in-person visits for more serious conditions. The public should be made aware that there is a growing body of evidence that supports the role of telemedicine in the management of serious health conditions. Telemedicine has been shown to be effective in managing chronic diseases, specifically, congestive heart failure, stroke, and chronic obstructive pulmonary disease [14]. While telemedicine should not replace in-person care entirely, it can supplement it and provide greater flexibility and convenience for patients and health care providers. Resistance to telemedicine for serious illnesses can be overcome by implementing a hybrid model that includes remote consultations, specialist consultations, remote monitoring, follow-up appointments, access to timely and appropriate care, and continuity of care [6]. This approach can increase patient trust and confidence in telemedicine, which can lead to higher adoption rates, resulting in better health outcomes and increased patient satisfaction.

This study found that the overall acceptance of telemedicine is quite substantial, with a substantial proportion of participants expressing a willingness to seek telemedicine consultations. However, it is also essential to note that a small minority of participants expressed that they were unlikely. These findings suggest that while the majority of individuals are receptive to telemedicine, there is still a segment of the population that may have reservations, concerns, or barriers to using telemedicine services. Identifying and addressing these concerns is critical to achieving broader telemedicine adoption.

Moreover, this study highlights notable regional discrepancies in the acceptance of telemedicine, with the Northeastern and Eastern regions exhibiting the greatest propensity for seeking remote medical consultations. This phenomenon can be attributed, in part, to the established correlation between regional economic prosperity and health care disparities within China. In China, high-quality medical resources are concentrated in the economically advanced Eastern region, while the Central and Western regions have a relative lack of health resources [15]. Furthermore, individuals residing in the affluent Eastern region tend to possess superior health outcomes [16]. Consequently, factors, such as health care infrastructure, public familiarity, and educational initiatives surrounding telemedicine, as well as local attitudes toward web-based health care delivery, might all play a role in shaping these regional variances. By recognizing and addressing these disparities, policy makers can develop tailored measures to enhance telemedicine adoption and ensure uniform access to quality health care across China.

Our finding also highlights the importance of addressing the regional disparities in telemedicine acceptance, particularly in poorer regions such as the Western area. These regions may potentially benefit the most from telemedicine due to their limited access to quality health care services. Enhancing acceptance of telemedicine in these areas can be achieved through various means, including increasing awareness and education about the benefits of telemedicine, providing accessible and user-friendly technology, and offering culturally sensitive telemedicine services that cater to the unique needs of diverse patient populations. Additionally, implementing policies that encourage the use of telemedicine, such as reimbursement models that support remote consultations, can help overcome financial barriers and promote greater adoption in resource-poor settings.

The findings of PLS-SEM on the relationships between various factors and the likelihood of seeking telemedicine consultations provide valuable insights into the key determinants influencing individuals’ intentions to use telemedicine. The analysis highlights that perceived usefulness and perceived ease of use play a significant and robust role in influencing the likelihood of seeking telemedicine consultations. It is worth noting that similar findings have been reported in other studies [17,18], underscoring the consistency of these factors as critical drivers of telemedicine adoption across various health care contexts. These consistent findings emphasize the importance of addressing perceived usefulness and ease of use when designing and promoting telemedicine services to ensure their acceptance and effectiveness among a diverse patient population.

This study also provides important insights into the varying degrees of acceptance among participants to use telemedicine for different types of illnesses. Notably, only a small proportion, representing 16.4% (n=165) of the participants, indicated a likelihood of seeking telemedicine for all types of illnesses. This finding suggests that while telemedicine is gaining acceptance as a viable option for medical consultation, there remains a significant portion of the population who may not be fully comfortable or confident in using telemedicine for a comprehensive range of health concerns. Respiratory issues emerged as a primary area of interest among participants, with a significant number indicating their likelihood to use telemedicine for such conditions. This heightened interest in telemedicine for respiratory issues could be attributed to the fact that respiratory problems, such as common colds and seasonal allergies, are highly prevalent and often less threatening compared to other serious health conditions. Patients may feel more comfortable seeking remote consultations for these common ailments, which they are more likely to encounter in their daily lives.

However, the results also reveal areas where telemedicine adoption may face challenges. For instance, a relatively low proportion of participants reported their likelihood to seek telemedicine for reproductive health issues. This suggests that individuals may have reservations or concerns related to the sensitive nature of reproductive health concerns and the preference for in-person consultations in this context. Likewise, a relatively small percentage expressed a willingness to use telemedicine for cancer-related matters. Cancer is a complex and serious health condition, and individuals may feel more comfortable receiving such diagnoses and treatments in traditional health care settings, where physical examinations and emotional support are readily available.

The results regarding participants’ preferences for the platform used in telemedicine consultations provide valuable insights into the communication methods they find most suitable and comfortable. First, it is noteworthy that many expressed a preference for video chat as their most favored platform for telemedicine consultations. Video chat offers the advantage of face-to-face interaction, allowing health care providers to visually assess patients and create a more personal connection. This preference may reflect the desire for a more comprehensive and visual consultation experience, which can be particularly important for conditions that require visual examination. Second, text chat emerged as the second most preferred platform. Text chat offers a written communication channel, which can be beneficial for discussing medical concerns, sharing information, and seeking advice without the need for real-time interaction. Text chat offers the advantage of enabling patients to furnish written descriptions of their medical concerns, thereby ensuring important details are accurately conveyed to health care providers.

### Limitations

This study shares the same constraints commonly associated with web-based surveys. Aside from socially desirable responses, this survey particularly tends to draw participants with computer literacy and a notable interest or predisposition toward the subject matter. Second, this study is constrained by an uneven distribution of samples, as it lacked adequate representation from the Southwestern and Northwestern regions. China’s Southwestern and Northwestern regions have relatively lower income compared to the higher-income Eastern and Coastal regions. Therefore, careful interpretation is necessary, and the findings may not be applicable to the general population. In particular, further research is required to explore and evaluate TAMs in resource-constrained environments.

In general, Cronbach α>0.7, CR>0.7, and AVE≥0.5 are considered acceptable benchmarks for validity and reliability [19-21]. In this study, the items concerning perceived usefulness and ease of use of telemedicine exhibited Cronbach α values lower than 0.7 and AVE values lower than 0.5, although the CR value is of acceptable value. While the general benchmark for Cronbach α is often set at 0.7 or higher for internal consistency, values slightly below this threshold can still be acceptable. Particularly in psychological research, a Cronbach α value of 0.5 was deemed acceptable or reliable [22-24]. Regarding the AVE values, both item values ranged between 39% and 41%, which are below the recommended level of 0.5. Nevertheless, it has been rationalized that the AVE may be a more conservative estimate of the validity of the measurement model [25], and based on CR alone, the researcher may conclude that the convergent validity of the construct is adequately reliable [26]. The CR for both perceived usefulness and ease of use of telemedicine ranges from 0.73 to 0.76, surpassing the recommended threshold of 0.60 [10,25]. Hence, the internal reliability of both measurement items could be deemed adequately acceptable.

### Conclusions

This study’s results highlight a generally positive attitude toward telemedicine, with a substantial proportion of individuals expressing a willingness to seek telemedicine consultations. However, it also underscores the need to address the concerns of those who remain hesitant or unlikely to use telemedicine. The regional differences in acceptance emphasize the importance of tailoring telemedicine implementation strategies to specific geographic areas to ensure widespread access to this valuable health care resource. While there is strong interest in using telemedicine for respiratory, skin, and musculoskeletal issues, health care providers and policy makers should be mindful of the lower willingness to adopt telemedicine for reproductive health and cancer-related concerns. Addressing patient concerns, ensuring privacy and security, and offering appropriate support and guidance in these areas could be essential for promoting broader telemedicine adoption across diverse health care needs. The findings from this research can provide valuable insights for government agencies, telemedicine implementers, and policy makers. They can use this information to comprehend the critical factors influencing the behavior of prospective telemedicine users and craft precise strategies and policies to facilitate its widespread adoption effectively.

## Appendix

Multimedia Appendix 1 Questionnaire.

Multimedia Appendix 2 CHERRIES (Checklist for Reporting Results of Internet E-Surveys) checklist.

## Abbreviations

AVE: average variance extracted
CR: composite reliability
PLS-SEM: partial least squares structural equation modeling
TAM: technology acceptance model
