# Supplementary material for: Perception and Acceptance of Telemedicine Use in Health Care Among the General Public in China: Web-Based Cross-Sectional Survey
Source: J Med Internet Res. 2024 Jul 16;26:e53497. doi: 10.2196/53497 (PMC11289571; doi:10.2196/53497)
Supplement: Multimedia Appendix 1 [file jmir_v26i1e53497_app1.docx]

**Telemedicine services post-COVID-19 pandemic:**

**meeting the needs of general public in China**

**Section A: General information**

| 1 | Age | __________ years old |
| --- | --- | --- |
| 2 | Gender | [ 1 ] Male  [ 2 ] Female |
| 3 | Highest educational level | [ 1 ] Primary school and below  [ 2 ] Secondary school  [ 3 ] High school/technical school  [ 4 ] Bachelor  [ 5 ] Masters/PhD |
| 4 | Family annual income (Chinese Yuan, CNY) | [ 1 ] <50,000  [ 2 ] 50,000 – 100,000  [ 3 ] 100,001 – 150,000  [ 4 ] 150,001 – 200,000  [ 5 ] 250,001 – 300,000  [ 6 ] >300,000 |
| 5 | Birthplace | [ 1 ] Urban  [ 2 ] Rural |
| 6 | Region | [ 1 ] Northern  [ 2 ] Northeastern  [ 3 ] Eastern  [ 4 ] Southern Central  [ 5 ] Southwestern  [ 6 ] Northwestern |

**Section B Perceived usefulness**

|  |  | Strongly agree | Agree | Disagree | Strongly disagree |
| --- | --- | --- | --- | --- | --- |
| 1. | Telemedicine is essential for use during public health emergencies (e.g., pandemics, infectious disease outbreaks, flooding etc) | [ 1 ] | [ 2 ] | [ 3 ] | [ 4 ] |
| 2. | Telemedicine is essential for use during the time of no public health emergencies | [ 1 ] | [ 2 ] | [ 3 ] | [ 4 ] |
| 3. | Telemedicine visits are cost savings compared to conventional way of seeking medical care. | [ 1 ] | [ 2 ] | [ 3 ] | [ 4 ] |
| 4. | Telemedicine can equally provide quality medical service as the traditional face-to-face consultation | [ 1 ] | [ 2 ] | [ 3 ] | [ 4 ] |

**Section C: Perceived ease of use**

|  |  | Strongly agree | Agree | Disagree | Strongly disagree |
| --- | --- | --- | --- | --- | --- |
| 1. | Telemedicine is an effective way to improve access to health services | [ 1 ] | [ 2 ] | [ 3 ] | [ 4 ] |
| 2. | Telehealth practitioners can provide medical services across geographic borders | [ 1 ] | [ 2 ] | [ 3 ] | [ 4 ] |
| 3. | Telemedicine can enable providers to deliver health services to patients at remote locations | [ 1 ] | [ 2 ] | [ 3 ] | [ 4 ] |
| 4. | Telemedicine can circumvent hospital visits of non-critical illnesses | [ 1 ] | [ 2 ] | [ 3 ] | [ 4 ] |
| 5. | Telemedicine can help to alleviate hospital congestion | [ 1 ] | [ 2 ] | [ 3 ] | [ 4 ] |

**Section D: Behavioral intention**

| 1 | How likely are you to seek telemedicine consultation if it is available to you? | [ 1 ] Very likely  [ 2 ] Likely  [ 3 ] Unlikely  [ 4 ] Strongly unlikely | |
| --- | --- | --- | --- |
| 2 | What types of illnesses do you think you will seek telemedicine consultation? | Yes | No |
| 2.1 | All illnesses | [ 1 ] | [ 2 ] |
| 2.2 | Respiratory (e.g. asthma, bronchitis, tuberculosis) | [ 1 ] | [ 2 ] |
| 2.3 | Muscoloskeletal (e.g arthritis, rheumatism, backache) | [ 1 ] | [ 2 ] |
| 2.4 | Skin diseases | [ 1 ] | [ 2 ] |
| 2.5 | Cardiovascular diseases (heart problem, stroke | [ 1 ] | [ 2 ] |
| 2.6 | Circulatory (hypertension) | [ 1 ] | [ 2 ] |
| 2.7 | Endocrine (e.g tyroid disease, diabetes, lupus | [ 1 ] | [ 2 ] |
| 2.8 | Neurologic (migraine, sclerosis, epilepsy) | [ 1 ] | [ 2 ] |
| 2.9 | Psychological (depression, anxiety and other emotional issue) | [ 1 ] | [ 2 ] |
| 2.10 | Digestive diseases (trouble with mouth, gums, ulcers, indigestion, diarrhea, constipation) | [ 1 ] | [ 2 ] |
| 2.11 | Cancer | [ 1 ] | [ 2 ] |
| 2.12 | Infectious diseases | [ 1 ] | [ 2 ] |
| 2.13 | Reproductive health and related diseases. | [ 1 ] | [ 2 ] |
| 3 | What is your MOST preferred platform for telemedicine consultation? | [ 1 ] Telephone  [ 2 ] Video chat  [ 3 ] Text chat  [ 4 ] Email | |
