# Supplementary material for: Perception and Acceptance of Telemedicine Use in Health Care Among the General Public in China: Web-Based Cross-Sectional Survey
Source: J Med Internet Res. 2024 Jul 16;26:e53497. doi: 10.2196/53497 (PMC11289571; doi:10.2196/53497)
Supplement: Multimedia Appendix 2 [file jmir_v26i1e53497_app2.pdf]

Checklist for Reporting Results of Internet E-Surveys (CHERRIES)

Checklist Item	***Explanation***	***Page Number***
Describe survey design	Describe target population, sample frame. Is the sample a convenience sample? (In "open" surveys this is most likely.)<br><br>*A nationwide cross-sectional survey using an online questionnaire was conducted from 30 August 2021 to 1st May 2022. A convenient sampling method was used.*	Pg 3<br>Line 113-114
IRB approval	Mention whether the study has been approved by an IRB.<br><br>*This study was implemented according to the principles of the Declaration of Helsinki and granted ethical approval by the Fujian Medical University Research Ethics Committee, China (Approval: FJMU 2022 No.64).*	Pg 5<br>Line 121
Informed consent	Describe the informed consent process. Where were the participants told the length of time of the survey, which data were stored and where and for how long, who the investigator was, and the purpose of the study?<br><br>*Informed consent involves providing participants with information sheets, informing them about the survey's timeframe, the estimated time required to complete it, and assuring them of the anonymity of their responses. The participants' information sheet also contains details about the investigators and the institution conducting the survey.*<br><br>*Data is securely stored on a cloud platform accessible only to the principal investigator.*	PG 6<br><br>Line 215<br><br>Line 223
Data protection	If any personal information was collected or stored, describe what mechanisms were used to protect unauthorized access.<br><br>*The survey did not collect any identifying information from participants.*	PG 6<br><br>Line 222
Development and testing	State how the survey was developed, including whether the usability and technical functionality of the electronic questionnaire had been tested before fielding the questionnaire.<br><br>*The study used electronic questionnaire.*	PG 3

	A nationwide cross-sectional survey using an online questionnaire was conducted from 30 August 2021 to 1st May 2022<br><br>*Questionnaire is self-developed.*<br><br>*Before the survey commenced, the questionnaire underwent content validation by a panel of experts in the field and was subsequently pilot tested with the general public before its official dissemination.*	Line 115<br><br>PG 5<br><br>Line182
Open survey versus closed survey	An "open survey" is a survey open for each visitor of a site, while a closed survey is only open to a sample which the investigator knows (password-protected survey).<br><br>*A nationwide cross-sectional open survey using an online questionnaire was conducted from 30 August 2021 to 1st May 2022.*	PG 3<br><br>Line 113
Contact mode	Indicate whether or not the initial contact with the potential participants was made on the Internet. (Investigators may also send out questionnaires by mail and allow for Web-based data entry.)<br><br>*Firstly, the research team advertise and disseminate the survey with colleagues, students, and their network contacts.*	PG 3<br><br>Line 126
Advertising the survey	How/where was the survey announced or advertised? Some examples are offline media (newspapers), or online (mailing lists – If yes, which ones?) or banner ads (Where were these banner ads posted and what did they look like?). It is important to know the wording of the announcement as it will heavily influence who chooses to participate. Ideally the survey announcement should be published as an appendix.<br><br>*Further, snowball sampling technique was used to enlist more participants across all provinces in mainland China, where individuals who received the survey link were encouraged to pass on the invitations to their own network members.*	PG 3<br><br>Line 128
Web/E-mail	State the type of e-survey (eg, one posted on a Web site, or one sent out through e-mail). If it is an e-mail survey, were the responses entered manually into a database, or was there an automatic method for capturing responses?	PG 3

	The study used Wenjuanxing, a widely accepted online questionnaire survey platform in China for data collection. The survey was disseminated mainly utilizing WeChat, China's largest social media platform. Responses were automatically captured by the survey platform and transfer to SPSS for analysis.	Line 115-118
Context	Describe the Web site (for mailing list/newsgroup) in which the survey was posted. What is the Web site about, who is visiting it, what are visitors normally looking for? Discuss to what degree the content of the Web site could pre-select the sample or influence the results. For example, a survey about vaccination on a anti-immunization Web site will have different results from a Web survey conducted on a government Web site<br><br>*The survey was distributed using a convenient sampling method and snowball technique, where participants shared the survey with their network contacts. No website was involved in the dissemination process.*	PG 3<br><br>Line 128
Mandatory/voluntary	Was it a mandatory survey to be filled in by every visitor who wanted to enter the Web site, or was it a voluntary survey?<br><br>*The participants were informed that their participation was voluntary.*	PG 6<br>Line 219
Incentives	Were any incentives offered (eg, monetary, prizes, or non-monetary incentives such as an offer to provide the survey results)?<br><br>*The participants were informed that their participation was voluntary, and no incentives, whether monetary or non-monetary, were provided.*	PG 6<br>Line 219
Time/Date	In what timeframe were the data collected?<br><br>*A nationwide cross-sectional open survey using an online questionnaire was conducted from 30 August 2021 to 1st May 2022.*	PG 3<br>Line 113-114
Randomization of items or questionnaires	To prevent biases items can be randomized or alternated.<br><br>*Question items were not randomized.*	-
Adaptive questioning	Use adaptive questioning (certain items, or only conditionally displayed based on responses to other items)	-

	to reduce number and complexity of the questions.<br><br>*We did not use adaptive questioning*	
Number of Items	What was the number of questionnaire items per page? The number of items is an important factor for the completion rate.<br><br>*The survey question is in one continuous page.*	-
Number of screens (pages)	Over how many pages was the questionnaire distributed? The number of items is an important factor for the completion rate.<br><br>*The survey question is in one continuous page.*	-
Completeness check	It is technically possible to do consistency or completeness checks before the questionnaire is submitted. Was this done, and if “yes”, how (usually JAVAScript)? An alternative is to check for completeness after the questionnaire has been submitted (and highlight mandatory items). If this has been done, it should be reported. All items should provide a non-response option such as “not applicable” or “rather not say”, and selection of one response option should be enforced.<br><br>*All questions in the questionnaire require a response; participants must fill them out before proceeding to the next page. As a result, all responses received through the survey platform are complete.*	PG 5<br><br>Line 180
Review step	State whether respondents were able to review and change their answers (eg, through a Back button or a Review step which displays a summary of the responses and asks the respondents if they are correct).<br><br>*Respondents were unable to review and change the answer.*	-
Unique site visitor	If you provide view rates or participation rates, you need to define how you determined a unique visitor. There are different techniques available, based on IP addresses or cookies or both.	-

	There is no view rates in the survey platform to determine survey engagement.	
View rate (Ratio of unique survey visitors/unique site visitors)	Requires counting unique visitors to the first page of the survey, divided by the number of unique site visitors (not page views!). It is not unusual to have view rates of less than 0.1 % if the survey is voluntary.<br><br>*As this is an open survey, unfortunately the system did not capture visitor.*	-
Participation rate (Ratio of unique visitors who agreed to participate/unique first survey page visitors)	Count the unique number of people who filled in the first survey page (or agreed to participate, for example by checking a checkbox), divided by visitors who visit the first page of the survey (or the informed consents page, if present). This can also be called "recruitment" rate.<br><br>*Information does not capture by the survey platform.*	-
Completion rate (Ratio of users who finished the survey/users who agreed to participate)	The number of people submitting the last questionnaire page, divided by the number of people who agreed to participate (or submitted the first survey page). This is only relevant if there is a separate "informed consent" page or if the survey goes over several pages. This is a measure for attrition. Note that "completion" can involve leaving questionnaire items blank. This is not a measure for how completely questionnaires were filled in. (If you need a measure for this, use the word "completeness rate".)<br><br>*Information does not capture by the survey platform.*	-
Cookies used	Indicate whether cookies were used to assign a unique user identifier to each client computer. If so, mention the page on which the cookie was set and read, and how long the cookie was valid. Were duplicate entries avoided by preventing users access to the survey twice; or were duplicate database entries having the same user ID eliminated before analysis? In the latter case, which entries were kept for analysis (eg, the first entry or the most recent)?<br><br>*Duplicate entries can be detected, and individuals are unable to answer more than once.*<br><br>*The survey platform prevents multiple responses by the same individual by disallowing users with the same IP address from accessing the survey more than once.*	PG 3<br>Line 116-118

IP check	Indicate whether the IP address of the client computer was used to identify potential duplicate entries from the same user. If so, mention the period of time for which no two entries from the same IP address were allowed (eg, 24 hours). Were duplicate entries avoided by preventing users with the same IP address access to the survey twice; or were duplicate database entries having the same IP address within a given period of time eliminated before analysis? If the latter, which entries were kept for analysis (eg, the first entry or the most recent)?<br><br>*The survey platform prevents multiple responses by the same individual by disallowing users with the same IP address from accessing the survey more than once.*	PG 3<br>Line 116-118
Log file analysis	Indicate whether other techniques to analyze the log file for identification of multiple entries were used. If so, please describe.<br><br>*Not applicable.*	-
Registration	In "closed" (non-open) surveys, users need to login first and it is easier to prevent duplicate entries from the same user. Describe how this was done. For example, was the survey never displayed a second time once the user had filled it in, or was the username stored together with the survey results and later eliminated? If the latter, which entries were kept for analysis (eg, the first entry or the most recent)?<br><br>*Our survey is an open survey.*	-
Handling of incomplete questionnaires	Were only completed questionnaires analyzed? Were questionnaires which terminated early (where, for example, users did not go through all questionnaire pages) also analyzed?<br><br>*There is not incomplete responses.*	-
Questionnaires submitted with an atypical timestamp	Some investigators may measure the time people needed to fill in a questionnaire and exclude questionnaires that were submitted too soon. Specify the timeframe that was used as a cut-off point, and describe how this point was determined.<br><br>*Unfortunately, the survey did not set cut-off point frame on duration of responses.*	-

Statistical correction	*Indicate whether any methods such as weighting of items or propensity scores have been used to adjust for the non-representative sample; if so, please describe the methods.*<br><br>*Not applicable.*	-

This checklist has been modified from Eysenbach G. Improving the quality of Web surveys: the Checklist for Reporting Results of Internet E-Surveys (CHERRIES). J Med Internet Res. 2004 Sep 29;6(3):e34 [erratum in J Med Internet Res. 2012; 14(1): e8.]. Article available at https://www.jmir.org/2004/3/e34/; erratum available https://www.jmir.org/2012/1/e8/. Copyright ©Gunther Eysenbach. Originally published in the Journal of Medical Internet Research, 29.9.2004 and 04.01.2012.

This is an open-access article distributed under the terms of the Creative Commons Attribution License (https://creativecommons.org/licenses/by/2.0/), which permits unrestricted use, distribution, and reproduction in any medium, provided the original work, first published in the Journal of Medical Internet Research, is properly cited.
